# Employee perception of managers’ attitudes towards older workers is associated with risk of loss of paid work before state pension age: prospective cohort study with register follow-up

**DOI:** 10.1007/s10433-022-00720-3

**Published:** 2022-08-10

**Authors:** Annette Meng, Emil Sundstrup, Lars L. Andersen

**Affiliations:** National Research Centre for the Working Environment, Lersø Parkallé 105, 2100 Copenhagen, Denmark

**Keywords:** Senior workers, Retention of workers, Social psychology, Occupational psychology, Ageism

## Abstract

It is increasingly urgent to retain older workers in the workforce. In the present study, we analysed the prospective associations between employees’ perceptions of their managers’ attitudes towards older workers, and of having experienced age discrimination in the labour market with the risk of loss of paid work before the state pension age. Questionnaire data from 10,320 currently employed workers aged 50 + on perceptions of managers’ attitudes towards older workers and perceived age discrimination were collected at baseline in the SeniorWorkingLife study. Data on labour market affiliation were obtained from national registers at baseline and two-year follow-up. Results show that the perception of negative attitudes was prospectively associated with an increased risk of loss of paid work for three of the five negative attitudes “older workers create conflicts, their qualifications are outdated, and they cannot keep up with the pace and development”. Perception of positive attitudes was prospectively associated with a reduced risk of loss of paid work. The perception of age discrimination was prospectively associated with an increased risk of loss of paid work. The results strengthen existing evidence on associations between ageism and labour market attachment, by applying a longitudinal design and including actual change in labour market participation. However, some negative attitudes may be more detrimental to the older workers’ labour market participation. Employees’ positive perceptions of managers’ attitudes reduced the risk. Good relations between employees and managers appear to be important for retaining older workers in the labour market.

## Introduction

Even though the participation rate of older workers in the workforce is anticipated to increase, a decline in labour supply is forecasted (EU 2017). To counteract this trend, particularly as the workforce ages, the retention of older workers becomes increasingly important. Therefore, it is essential to accumulate knowledge on factors associated with the labour market attachment among older workers.

Older workers may lose labour market attachment through retirement, sickness absence, disability pension, and unemployment. Many factors have been linked to early or late withdrawal from the labour market (Fisher et al. 2016; Meng et al. 2020). Important factors include health status (de Wind et al. 2014, 2016; Friis et al. 2007; Nilsson et al. 2011; Sewdas et al. 2017; Virtanen et al. 2014; Wahrendorf et al. 2017), the financial situation of the worker (Friis et al. 2007), influence from the family or the family situation (An et al. 2004; de Wind et al. 2014; Friis et al. 2007; Nilsson et al. 2011; Reeuwijk et al. 2013), leisure time preferences (Carlstedt et al. 2018; Reeuwijk et al. 2013), and physical and mental work demands (Andersen et al. 2019; Carlstedt et al. 2018; Sejbaek et al. 2013; van den Berg et al. 2010). Psychosocial factors at work have also been highlighted as important for the labour market attachment, including recognition (Carr et al. 2016; Sundstrup et al. 2018; Wilson et al. 2020), social support (Tuckett et al. 2015; van den Berg et al. 2010; Wilson et al. 2020), and job satisfaction (van den Berg et al. 2010; Wilson et al. 2020), and more specifically, to be related to sickness absence and disability pension (Knardahl et al. 2017; Sundstrup et al. 2018). Perceived negative attitudes towards older workers and age discrimination may be interpreted as a lack of recognition and social support by the older workers and may reduce their job satisfaction, and thus contribute to pushing the older workers towards early withdrawal from the labour market (Carr et al. 2016; van den Berg et al. 2010; Wilson et al. 2020). However, ageism or perceived ageism may influence older workers’ labour market attachment through various mechanisms. It has also been argued that negative attitudes towards older workers may be internalised by the older workers themselves and become self-stereotypes. This could further have a negative impact on their self-efficacy and end up as self-fulling prophecies (Weber et al. 2019), making the older workers unsure about their performance and more likely to give up working when perhaps facing a decline in work ability. The self-fulfilling prophecy could also have a negative impact on the actual performance of the older workers, perhaps making the management less motivated to retain them, increasing their risk of being made redundant. Furthermore, if the management holds negative attitudes towards older workers, they may be less motivated to put efforts into retaining the older workers, increasing the risk that they are pushed out of the labour market. In addition, Nilsson et al. (2016) found that feeling discriminated against because of age at the workplace, and the managers’ attitudes towards seniors were associated with self-rated health, which is further associated with the belief whether one can work until the age of 65. All in all, both the perception of having been exposed to age discrimination and that the management holds negative attitudes towards older workers, whether real or not, are likely to contribute to older workers’ early withdrawal from the labour market.

Research has identified a long list of negative stereotypes and beliefs about older workers, including that older workers are more resistant to change, less adaptable and flexible, less motivated and productive, are harder to train and have lower ability to learn, are more costly and have shorter job tenure (Posthuma and Campion 2007). Cross-sectional findings indicate that there is an association between the experience of ageism and the desire to or intention to retire early (Bayl-Smith and Griffin 2014; Nilsson et al. 2011; Schermuly et al. 2014; Snape and Redman 2003; Soidre 2005; Thorsen et al. 2012; Zaniboni 2015). These findings are supported by a prospective analysis by Thorsen et al. (2016), showing that perceived age discrimination was associated with early retirement. In addition, research has found that negative attitudes towards older workers affect employers’ hiring intentions and decisions (Fasbender and Wang 2016; Kadefors and Hanse 2012; Lu et al. 2011; Porcellato et al. 2010; Turek and Henkens 2020), thereby making it more difficult for older workers to change jobs or to re-enter the labour market if they lose their jobs. Together these findings provide strong indications that negative attitudes and age discrimination could represent a barrier to older workers’ labour market attachment. Nevertheless, the majority of the research in the field is based on cross-sectional analyses and measures retirement intentions rather than actual labour market attachment. As mentioned earlier, multiple factors influence the timing of the withdrawal from the labour market (Fisher et al. 2016; Meng et al. 2020) therefore, intentions to retire early or late may not necessarily predict actual future labour market attachment. Therefore, change in actual labour market attachment presents a stronger outcome measure in this area of research. Also, the need for research that extends beyond the cross-sectional design has been emphasised (Harris et al. 2018) and will help provide more solid evidence for the association between the experience of ageism and actual labour market attachment.

Furthermore, managers and employers are generally found to hold more positive than negative attitudes towards older workers (Jenkins and Poulston 2014; Jensen and Møberg 2012; Kluge and Krings 2008; Meng et al. 2021; Van Dalen et al. 2009). The positive stereotypes and beliefs include that older workers are committed, loyal, honest, trustworthy, more stable, dependable, and less likely to miss work or turnover quickly (Posthuma and Campion 2007). However, to the authors’ knowledge, so far no studies have systematically explored whether positive perceptions of managers’ attitudes towards older workers actually protect against older workers’ early withdrawal from the labour market. Interviews with persons who were willing to work until retirement age or beyond revealed that appreciation of the work done and being needed were factors motivating them to continue working (Proper et al. 2009) and that social connectedness and being appreciated were important benefits of continuing working (Reynolds et al. 2012). It is plausible that perceptions of positive attitudes towards older workers contribute to them feeling appreciated and socially connected and motivate them to stay at their workplace for as long as possible. Similarly, it can be expected that managers who do have positive attitudes towards older workers are more likely to put efforts into retaining their older employees, supporting prolonged labour market attachment.

In the present study, we analysed the prospective association of perceived positive and negative attitudes towards older workers and the perceived experience of age discrimination with the risk of loss of paid work. Perceptions of negative attitudes towards older workers are more general and may be perceived to apply to oneself to a varying degree, while the perception of having experienced age discrimination implies that one personally was the target of a negative act. Therefore, we decided to include both perceptions of managers’ attitudes towards older workers and perceived age discrimination in the analyses.

We tested the following three hypotheses:

1. Participants who believe that their managers hold negative attitudes towards older workers have an increased risk of loss of paid work after two-year follow-up.
2. Participants who believe that their managers hold positive attitudes towards older workers have a reduced risk of loss of paid work after two-year follow-up.
3. Participants who perceive to have experienced age discrimination in the labour market have an increased risk of loss of paid work after two-year follow-up.

## Method

### Design and sample

We estimate the prospective associations of questionnaire data on perceived attitudes towards older workers and perceived experience of age discrimination with labour market participation from national registers.

The SeniorWorkingLife study is registered in ClinicalTrials.gov (Identifier: NCT03634410) as a cohort study, and the protocol is published as open-access (Andersen and Sundstrup 2019). The baseline data were collected from July to October 2018. At baseline, a total of 18,000 employed individuals aged 50 years or older were drawn as a probability sample by Statistics Denmark and invited to participate with a personal questionnaire link via e-Boks (online digital mailbox linked to the Danish social security number). Of the invited employed individuals, 56% completed the entire questionnaire. Model-assisted weights were applied in the analyses to ensure that the results were representative of the population (Andersen and Sundstrup 2019). Only participants who confirmed in the baseline questionnaire that they were currently employed and had responded to the questions regarding perceived attitudes towards older workers or experience of age discrimination in the baseline questionnaire were included in the analyses. Furthermore, to ensure that the participants had loss of paid work before the state pension age, the study population was truncated at a maximal age of 63 year at baseline in 2018. In 2018, the state pension age was 65 years, but this was raised to 66 years in 2020. Thus, participants aged 63 years could not reach more than 65 years at follow-up, i.e. before the state pension age. The total study sample included in the analyses was thereafter 10,320. Information about loss of paid work was obtained from a national register on labour market affiliation handled by Statistics Denmark at the time of the baseline questionnaire survey in 2018 and again at the time of the follow-up questionnaire survey in 2020. Because the analyses in connection to this article did not include questions from the survey at follow-up, no participants were lost to follow-up.

### Measurements

#### Perceived attitudes towards older workers

Perceived attitudes towards older workers were measured with the following question “Do you think the managers at your workplace think the following about older workers?” followed by a list of ten attitudes towards older workers; five positive and five negative (the ten attitudes are presented in Table 2). The list of attitudes was provided in random order, and the respondents were required to mark the attitudes they believed their managers had (multiple choice). The list of attitudes was formulated for the purpose of this study but was inspired by the literature in the field (Harris et al. 2018; Henkens 2005; Jensen and Møberg 2012; Posthuma and Campion 2007; Van Dalen et al. 2010).

#### Perceived experience of age discrimination

Perceived experience of age discrimination was measured with the following question “Have you experienced being discriminated against because of your age in the labour market?” with the following response options: “yes”, “no”, “do not know”, “do not wish to reply”. The question was formulated for the purpose of this study and aimed to capture the experience of age discrimination across various situations in the labour market, for example, hiring, during work, and dismissal.

#### Loss of paid work before state pension age

The criteria for paid work were as follows: the person should have been in paid work for at least 20 h per week in at least half of the time in the year up to the questionnaire survey *and* in paid work at least 20 h per week in the most recent month prior to the questionnaire survey. Loss of paid work was defined as persons who at the baseline questionnaire survey in 2018 fulfilled the criteria for paid work of at least 20 h per week and at the time of the follow-up questionnaire survey in 2020, no longer fulfilled these criteria. As mentioned above, the study population was truncated at the age of 63 at baseline, and therefore, the outcome variable is loss of paid work before the state pension age. Individuals with loss of paid work, thus, included individuals who had left the labour market either permanently or temporarily, or substantially reduced their working hours before state pension age. Therefore, we use the term “loss of paid work” rather than “exit from paid work”, “early retirement” or “loss of labour market attachment”.

### Control variables

Because these factors have been found to be associated with work ability, disability pension, and early withdrawal from the labour market (Calatayud et al. 2015; van den Berg et al. 2009; Venti and Wise 2015), we controlled for age (years), sex (male/female), education (unskilled, skilled, and further education), and lifestyle (body mass index (BMI: kg/m2), smoking status (No/Yes), and physical activity during leisure (four categories)).

### Statistics

Using logistic regression (PROC GLIMMIX, SAS version 9.4) with model-assisted weights, representative risk ratios for loss of paid work during 2-year follow-up period were modelled as a function of each predictor variable. The statistical model was adjusted for the before-mentioned control variables. The estimates are presented as risk ratios (RR) with 95% confidence intervals (CIs).

## Results

Demographic and lifestyle information about the participants are provided in Table 1. In addition, the results show that at two-year follow-up, 12% of the participants had lost paid work.

**Table 1:** Demographic and lifestyle information about the participants at baseline

	Mean (SD) or *n* (%)
Age (mean (SD))	55.8 (5.4)
Women (*n* (%))	4812 (48)
Men (*n* (%))	5508 (52)
Education (*n* (%))	
Unskilled manual worker	2043 (21)
Skilled manual worker	4389 (41)
Higher education	3888 (38)
Smoking (*n* (%) yes)	1963 (19)
BMI (*n* (%))	
< 18	57 (1)
18- < 25	4256 (42)
25- < 30	4056 (40)
30- < 35	1388 (13)
35- < 40	321 (3)
≥ 40	103 (1)
Leisure time activity (*n* (%))	
Seated	1494 (14)
Light exercise at least 4 h per week	6221 (61)
Sports or heavy physical activity at least 4 h per week	2358 (23)
Training and competing regularly and several times a week	203 (2)

When looking at the association between perceived attitudes toward older workers and loss of paid work, the results show that, in particular, the belief that the managers think that older workers create conflicts is associated with an increased risk of loss of paid work, but also that the qualifications of older workers are outdated and that older workers cannot keep up with the pace and development. The remaining two negative attitudes “Older workers are preoccupied with their retirement” and “Older workers should make room for the young” were not significantly associated with a higher risk of loss of paid work (see Table 2). The results thus partly support Hypothesis 1.

**Table 2:** Relative risk (95% confidence intervals) for loss of paid work at 2-year follow-up from agreeing that “The managers at your workplace think the following about older workers”

Do you think the managers at your workplace think the following about older workers?	Agree (%)	RR (95% CI)
Older workers’ experience and knowledge is a significant resource for the workplace	56	**0.70** (0.69–0.71)
Older workers are productive	17	**0.80** (0.79–0.82)
Older workers are flexible in regards to working hours	28	**0.89** (0.87–0.90)
Older workers are flexible in regards to work tasks	25	**0.90** (0.88–0.91)
Older workers are easy to cooperate with	18	**0.92** (0.91–0.94)
Older workers’ qualifications are outdated	6	**1.18** (1.15–1.22)
Older workers cannot keep up with the pace and development	11	**1.17** (1.14–1.20)
Older workers are preoccupied with their retirement	2	1.02 (0.97–1.08)
Older workers should make room for the young	4	0.98 (0.95–1.02)
Older workers create conflicts	2	**1.42** (1.37–1.48)
Don’t know	28	**1.22** (1.20–1.24)

*n* = 10,304. Analysis controlled for sex, age, education, and lifestyle (smoking, BMI, leisure-time physical activity). Significant RR’s are highlighted in bold

Regarding the positive attitudes, the results show that the belief that the managers hold positive attitudes towards older workers is significantly associated with a lower risk of loss of paid work at follow-up. Particularly, when the participants believe that the managers hold the opinion that the experience and knowledge of older workers are a significant resource for the workplace and that older workers are productive (see Table 2). Thus, the results provide support for Hypothesis 2.

Interestingly, as shown in Table 2, not knowing what the managers think about older workers is associated with a higher risk of loss of paid work.

Lastly, the results supported Hypothesis 3 by showing that participants who reported having experienced to be discriminated against because of their age in the labour market had a significantly higher risk of loss of paid work during the two-year follow-up period (see Table 3).

**Table 3:** Relative risk (95% confidence intervals) for loss of paid work at 2-year follow-up for participants who have and have not experienced age discrimination in the labour market

Have you experienced to be discriminated against because of your age in the labour market?	*n* (%)	RR (95% CI)
Yes	659 (4.8)	1.61 (1.58–1.65)
No	9622 (95.2)	1

Analysis controlled for sex, age, education, and lifestyle (smoking, BMI, leisure-time physical activity)

## Discussion

The belief that the managers at the workplace held three of the five negative attitudes towards older workers was significantly associated with an increased risk of loss of paid work. Thus, Hypothesis 1 was partly supported. Perceptions of all five positive attitudes towards older workers were significantly associated with a reduced risk of loss of paid work, providing support for Hypothesis 2. Lastly, results showed that the perception to have experienced age discrimination in the labour market was significantly associated with an increased risk of loss of paid work and thus provided support for Hypothesis 3.

### Perceived negative attitudes and loss of paid work

The results showed that the participants who believed their managers held the following three negative beliefs about older workers, had an increased risk of loss of paid work at two-year follow-up: “Older workers’ qualifications are outdated” which increased the risk by 18%, “Older workers cannot keep up with the pace and development” with a 17% increase, and “Older workers create conflicts” with a 42% increased risk of loss of paid work. The first two negative attitudes reflect negative expectations regarding the performance of older workers. Negative attitudes towards older workers may be internalised by the older workers themselves and become self-stereotypes. This could further have a negative impact on their self-efficacy and end up as self-fulling prophecies (Weber et al. 2019). This could lead to the older workers becoming unsure about their performance and more likely to give up working when faced with declining work ability. It is plausible that negative attitudes related to older workers’ performance are particularly salient for this mechanism. Perceived negative attitudes towards older workers’ performance may also be interpreted as a lack of recognition, which has been found to be associated with sickness absence and disability pension (Knardahl et al. 2017; Sundstrup et al. 2018). These possible internal mechanisms in the older workers can be expected to be independent of whether the perceived attitudes reflect the true attitudes of the managers or they are misinterpretations. At the same time, in some cases, the perceived negative attitudes may reflect the true attitudes of the managers, that they believe that the performance of the older employees is inferior. In such cases, the increased risk of loss of paid work may reflect less effort by these managers to retain the older workers.

The third negative attitude that was significantly associated with an increased risk of loss of paid work was “older workers create conflicts”. A qualitative study found that the attitudes of managers not only reflect stereotypes but are also based on concrete experiences with older workers. The authors describe how the managers told that older workers take on various roles, and one of these is “the conflict generating chiefs” based on the experiences that some older workers are stubborn and resist change, and create conflicts (Frøyland and Terjesen 2020). The perception that the managers believe that older workers create conflicts may reflect relationships between older employees and managers that are characterised by higher levels of conflict. This may be perceived as a lack of social support and related to reduced job-satisfaction among these older workers, which has been found to be associated with sickness absence and disability pension (Knardahl et al. 2017; Sundstrup et al. 2018). At the same time, if the managers do indeed perceive their older employees to be creating conflicts, they may be less motivated to put effort into retaining them.

Participants who believed that their managers had the following two negative beliefs about older workers, did not show an increased risk of loss of paid work: “Older workers are preoccupied with their retirement” and “Older workers should make room for the young”. Although these attitudes could be expected to be interpreted as a lack of recognition, Bayl-Smith and Griffin (2014) suggest that some older workers may decide to stay at work to differentiate themselves from stereotypes that insinuate that older workers are slowing down and ready for retirement. Along these lines, it could be that these two types of attitudes towards older workers are more likely to induce defiance in some older workers, motivating them to put effort into staying in their jobs and perhaps increase their self-efficacy.

Overall, the results provide some support for previous cross-sectional findings that negative attitudes towards older workers are associated with exit from the labour market (Bayl-Smith and Griffin 2014; Nilsson et al. 2011; Schermuly et al. 2014; Snape and Redman 2003; Soidre 2005; Thorsen et al. 2012; Zaniboni 2015). However, the results also indicate that it may be more complex and that some negative attitudes may be more detrimental to the older workers’ labour market participation. More research is needed to create a deeper understanding of the mechanisms through which these negative attitudes, whether perceived or real, have an impact on the labour market attachment of older workers.

### Perceived positive attitudes and loss of paid work

Perceptions of all five positive attitudes were associated with a reduced risk of loss of paid work at two-year follow-up. Thus, the results indicate that perceived positive attitudes towards older workers indeed do have a positive influence on the labour market participation of the older workers, supporting Hypothesis 2. The perceived positive attitudes that were associated with the largest reduction of the risk were “Older workers’ experience and knowledge is a significant resource for the workplace” which was associated with a 30% reduced risk and “Older workers are productive” with a 20% reduced risk. The belief that the managers hold these attitudes may lead to the older workers feeling appreciated, recognised, and that their accumulated experience and knowledge are needed at the workplace. These factors have previously been found to motivate older workers to stay in the workforce (Proper et al. 2009) and may have a positive impact on their self-confidence and self-efficacy. The belief that the managers hold positive attitudes may also reflect the real attitudes of the managers and the reduced risk of loss of paid work could be the result of more retention efforts on the behalf of the managers. Overall, the results suggest that good relations between employees and managers are important for the retention of older workers. The results also call for efforts to promote a positive image of older workers to support the development of positive attitudes towards older workers in both the older workers themselves and the management. Finally, the results indicate that it is advantageous when managers explicitly express their positive attitudes towards their older employees.

Interestingly, participants who did not know what their managers were thinking about older workers had an increased risk of loss of paid work. This finding could perhaps reflect unclear communication and actions on the behalf of the managers as well as a distance between the managers and the older workers, which may again be associated with the level of engagement of the older workers and the managers’ motivations to put effort into retaining the older workers. However, this is mere speculation, the data does not allow for further exploration of this association.

### Perceived age discrimination and loss of paid work

Lastly, the results showed that participants who perceived to have experienced age discrimination had a 61% increased risk of loss of paid work, providing support for Hypothesis 3. This result is in line with the prospective findings from Thorsen et al. (2016) that perceived age discrimination is associated with early retirement and provide further indications of a causal relationship between the perception of age discrimination and exit from the labour market among older workers. Nilsson et al. (2016) found that the perception of age discrimination was associated with self-rated health, which again was associated with beliefs about ones ability to work until the age of 65. Our results could reflect this mechanism and indicate that it may not only be related to the beliefs about one’s ability to continue working, but may predict actual change in future labour market attachment. The perception of age discrimination presented a larger risk of loss of paid work than the perceived negative attitudes. This probably reflects that the perceived experience of age discrimination implies that the older worker personally was the target of the perceived negative behaviour, while perceptions of negative attitudes towards older workers are more general and may be less personally relevant. In cases where the perception of having experienced age discrimination reflects actual acts of age discrimination, the loss of paid work may be an indication of a lack of efforts to retain the older workers at the workplace or maybe even active efforts to push them out of the workplace.

## Limitations and strengths of the study

Among the strengths of this study is the large and representative sample of older workers along with the prospective study design allowing for conclusions on the direction of the associations found. In addition, the outcome variable “loss of paid work” was drawn from a national register database and thus represents actual behaviour and not merely intentions to exit the labour market, which is the outcome variable in the majority of the existing studies. So, although causal inferences cannot be drawn from observational studies, the prospective associations combined with previous knowledge in the literature suggest that negative perceptions as well as perceptions of age discrimination are important factors that can push people out of the labour market.

Another strength of the study is the use of model-assisted weights in the analyses reducing the risk of bias due to differences in response rates and size between subgroups of the study population and thereby ensuring that the results are representative of the population.

A limitation is that we asked about perceived attitudes towards older workers. We therefore do not know to what extent the participants believed that the managers held these attitudes towards themselves personally. However, it is not unlikely that the perceptions of the managers’ attitudes reflect personal experiences with the managers. Regarding the experience of age discrimination, we asked directly about personal experiences of age discrimination; however, the question was formulated more broadly as “in the labour market”. We therefore cannot be certain that it was at the participants’ current workplace that they had experienced the age discrimination. Again, it is not unlikely that the majority of the participants had their current workplace in mind when responding to the question. Another limitation of using perceptions of age discrimination and of managers’ attitudes towards older workers is that we do not know to what extent it reflects the real attitudes and behaviour of the managers. It may be that the older workers in some cases misinterpreted attitudes and behaviours of the managers as ageist, perhaps due to negative self-concept, when in fact this was not the case.

A further limitation is that the questions on attitudes and age discrimination have not been psychometrically validated. Nevertheless, they have a sound base in the literature and thus reflect attitudes commonly included in this field of research.

Fortunately, only the minority of the participants reported their managers to hold negative attitudes towards older workers. However, this posed a limitation to which analyses were possible to perform due to reduced statistical power. It was, for example, not possible to explore differences between job groups or other subgroups.

A final limitation is that the outcome variable “loss of paid work” could not be divided into further categories and thus consisted of all participants, who were previously employed for at least 20 h per week and no longer fulfil these criteria after two-year follow-up. However, leaving the labour market or substantially reducing the working hours before the state pension age is likely to reflect push mechanisms, where particularly reduced work ability or unemployment lead to the loss of paid work. However, some may also have lost paid work due to voluntary early retirement.

## Conclusion

The results showed that employees’ negative perception of managers’ attitudes and perceived age discrimination increased the risk of loss of paid work after two-year follow-up. Thus, the results support earlier cross-sectional findings and strengthen existing evidence by including actual change in labour market participation and not only expectations regarding future labour market attachment. However, the results also indicate that the association may be more complex and that some negative attitudes may be more detrimental to the older workers’ labour market participation. In addition, the results showed that employees’ positive perception of managers’ attitudes reduced the risk of loss of paid work. The results, thus, call for efforts to promote a positive image of older workers to support the development of positive attitudes towards older workers in both the older workers themselves and the management. Lastly, the results indicate that it is advantageous when managers explicitly express their positive attitudes towards their older employees.

## Data Availability

The authors encourage collaboration and use of the data by other researchers. Data are stored on the server of Statistics Denmark, and researchers interested in using the data for scientific purposes should contact the project leader Prof. Lars L. Andersen, lla@nfa.dk.
